# Multiple Pregnancy (Quintuplets) and Treatment Considerations for Critical Ovarian Hyperstimulation Syndrome

**DOI:** 10.7759/cureus.99238

**Published:** 2025-12-14

**Authors:** Yu Horibe, Kei Koshimizu, Saki Minamisawa, Akiko Yamaguchi, Tsutomu Tabata

**Affiliations:** 1 Obstetrics and Gynecology, Tokyo Women's Medical University, Tokyo, JPN

**Keywords:** controlled ovarian stimulation, multiple pregnancy, ovarian hyperstimulation syndrome, polycystic ovary syndrome, quintuplets

## Abstract

Ovarian hyperstimulation syndrome (OHSS) associated with multiple gestation is often severe, necessitating a multidisciplinary approach. We report a case of OHSS that demonstrated a two-stage progression of severity, induced by controlled ovarian stimulation (COS) and a subsequent quintuplet pregnancy. This report details an exceptionally severe case of critical OHSS in a woman following COS that was profoundly exacerbated by quintuplets. The patient developed a life-threatening clinical course characterized by massive third-space fluid shifts, manifesting as significant ascites, pleural effusion, and subsequent hemodynamic instability. This condition necessitated aggressive multidisciplinary management. Therapeutic uterine evacuation was performed as a lifesaving measure for maternal stabilization, resulting in rapid clinical resolution. This case illustrates the pivotal importance of a coordinated multidisciplinary approach in managing these complex scenarios and underscores that pregnancy termination must be considered a definitive therapeutic intervention when maternal life is acutely compromised, despite the inevitable and significant delay to future fertility treatments.

## Introduction

Ovarian hyperstimulation syndrome (OHSS) is an iatrogenic condition that emerged with the advent of controlled ovarian stimulation (COS) in assisted reproductive technology. Its pathogenesis is multifactorial, involving the production of vasoactive substances, notably vascular endothelial growth factor (VEGF), leading to increased vascular permeability, subsequent intravascular dehydration, and a hypercoagulable state. OHSS is classified by severity, ranging from mild to severe, with severe cases often requiring hospitalization. Symptoms are diverse, and severe forms can present with nausea, abdominal distension, dyspnea, and circulatory failure [1]. We report a case of OHSS that progressed in two stages, associated with COS and multiple gestation, necessitating management in the ICU. We present this case with a review of the literature on its treatment and clinical course.

## Case presentation

**Figure 1:**
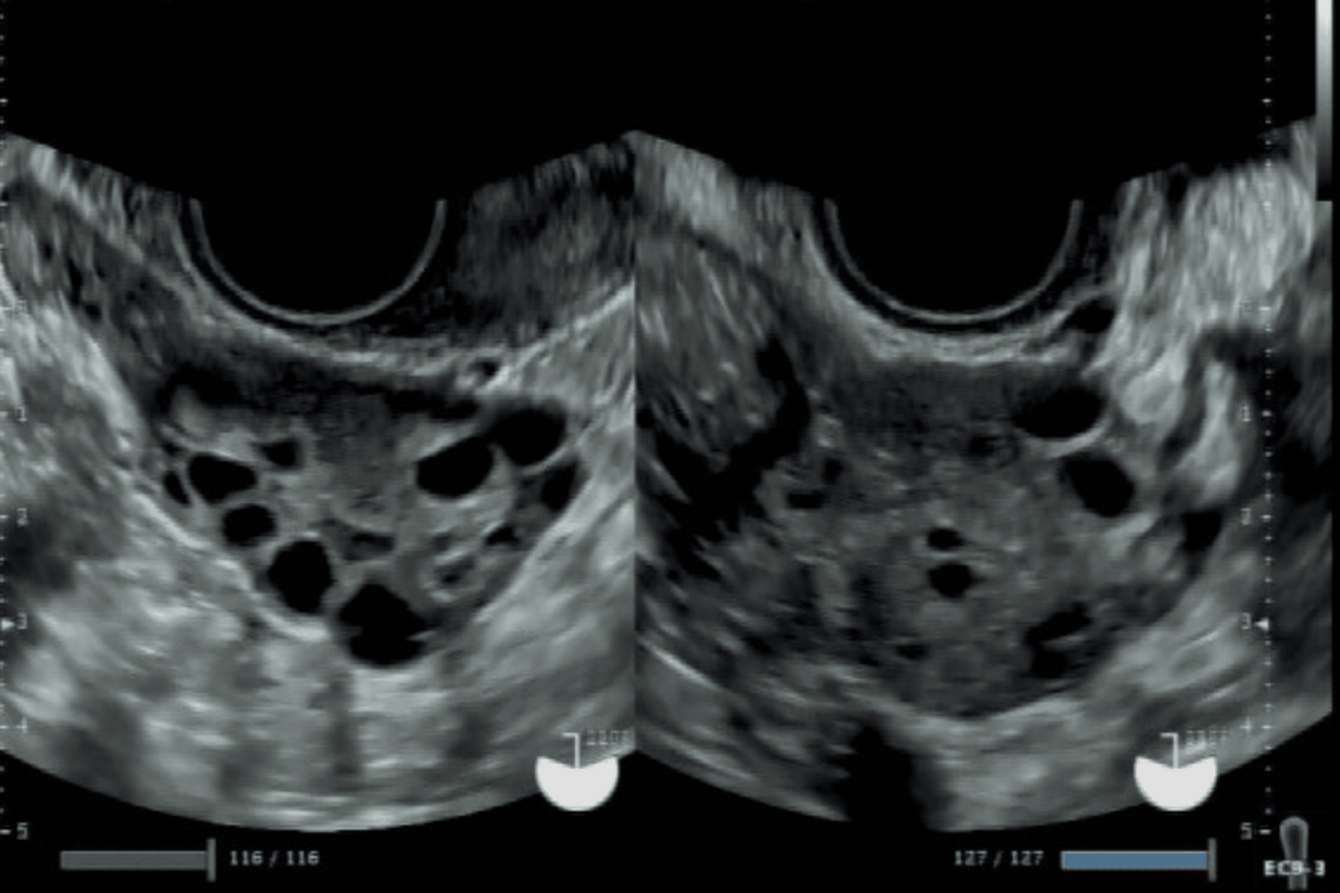
Transvaginal ultrasonography showing polycystic ovary morphology in both ovaries

The patient was a 29-year-old, nulligravid, nulliparous married woman desiring pregnancy who had been undergoing COS at another facility. She had no comorbidities. Transvaginal ultrasonography revealed polycystic ovary morphology, although she had not been formally investigated for or diagnosed with polycystic ovary syndrome (PCOS) (Figure 1). Her anti-Müllerian hormone (AMH) level was 2.78 ng/mL.

During the COS cycle preceding the onset of OHSS, she was started on follitropin alfa (recombinant follicle-stimulating hormone, rFSH) at 175 IU. Due to an insufficient ovarian response, the dosage was increased. Starting from day 3 of her menstrual cycle, she took letrozole 5 mg daily for five days. Additionally, from day 11 to day 22 of her menstrual cycle, the rFSH dose was increased to 225 IU, for a total of 1,575 IU administered. On day 18, when follicle measurements confirmed four follicles at 20 mm and two each at 16 mm and 15 mm, 10,000 IU of human chorionic gonadotropin (hCG) were administered, followed by intrauterine insemination.

From day 21, she experienced worsening lower abdominal pain, abdominal distension, dizziness, lightheadedness, and nausea, prompting a visit to her previous physician. At that time, her ovaries were enlarged (right: 14 × 11 cm; left: 19 × 14 cm), and she was diagnosed with severe OHSS and hospitalized. She received 2,000 mL of IV fluids daily and was discharged on day 25.

On day 31, she developed dyspnea and difficulty ambulating, leading to an emergency transfer to a higher-level facility. Despite treatment with IV fluids, dopamine hydrochloride, albumin transfusions, and 10,000 IU of heparin daily for massive ascites and pleural effusion, her symptoms, including vulvar edema and immobility, worsened, and her SpO₂ levels declined. Consequently, she was transferred to our university hospital on day 40.

**Figure 2:**
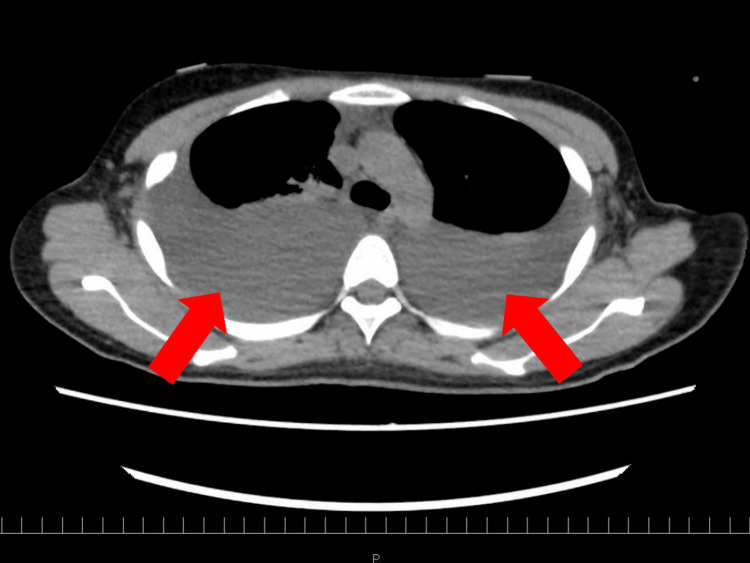
Non-contrast chest CT image at the time of admission showing large bilateral pleural effusions

Upon admission, examination revealed massively enlarged ovaries reaching the subphrenic space and bilateral pleural effusions (Figure 2).

Laboratory findings upon emergency admission were as follows: hemoglobin (Hb), 13.7 g/dL (reference range: 12.0-16.0); platelet count, 419,000/µL (150,000-350,000); hematocrit, 39.8% (35-43); albumin, 2.3 g/dL (3.8-5.1); total protein, 4.7 g/dL (6.5-8.2); and creatinine, 0.64 mg/dL (0.48-0.79).

**Figure 3:**
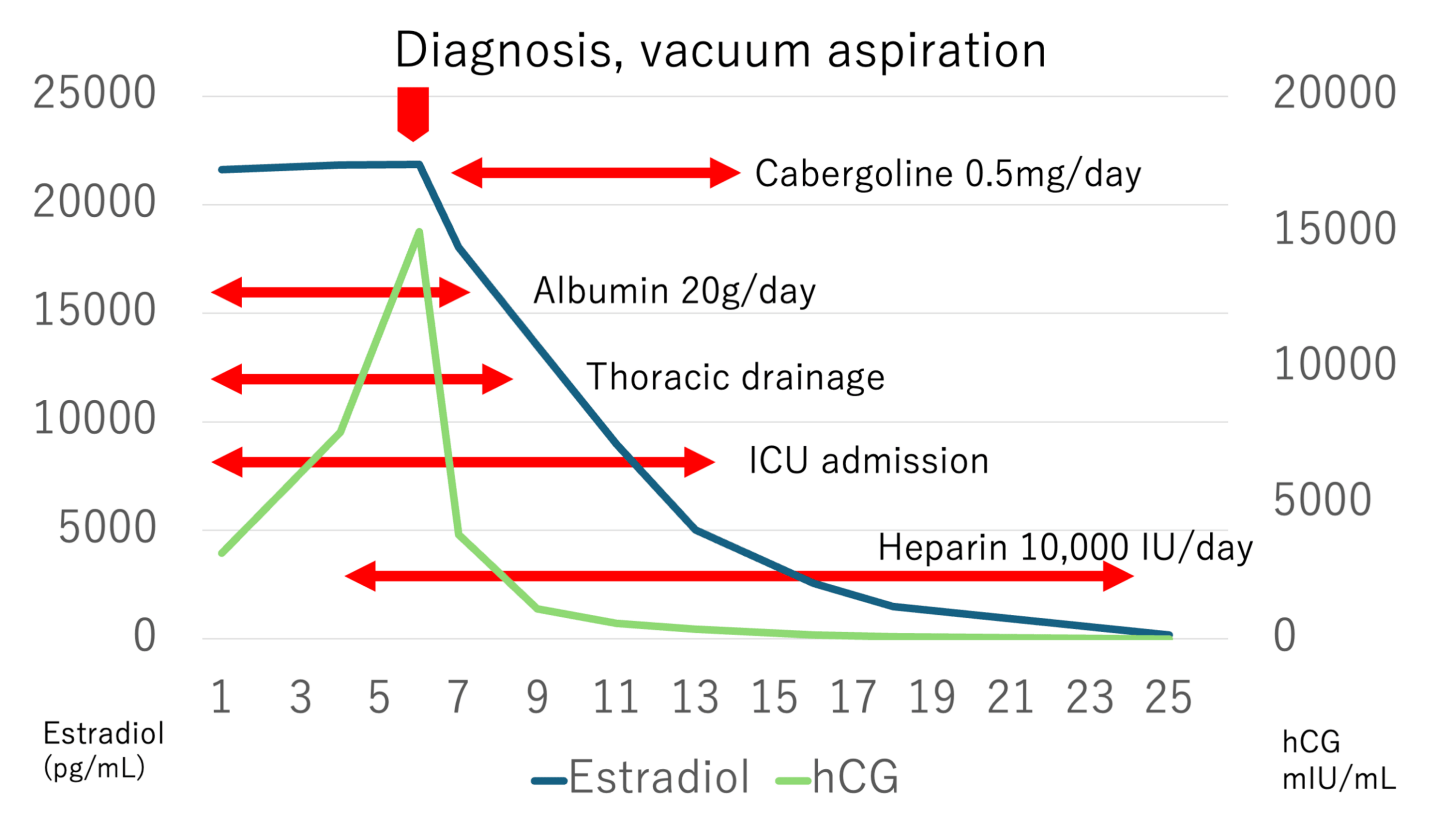
Changes in estradiol and hCG levels and clinical events after hospitalization Following vacuum aspiration, serum estradiol and hCG levels decreased, and the patient's symptoms gradually resolved. hCG, human chorionic gonadotropin

She was admitted to the ICU and received multidisciplinary treatment, including oxygen therapy, IV fluids, albumin transfusions, chest drainage, and anticoagulation therapy. Her clinical course after admission is summarized in Figure 3.

**Figure 4:**
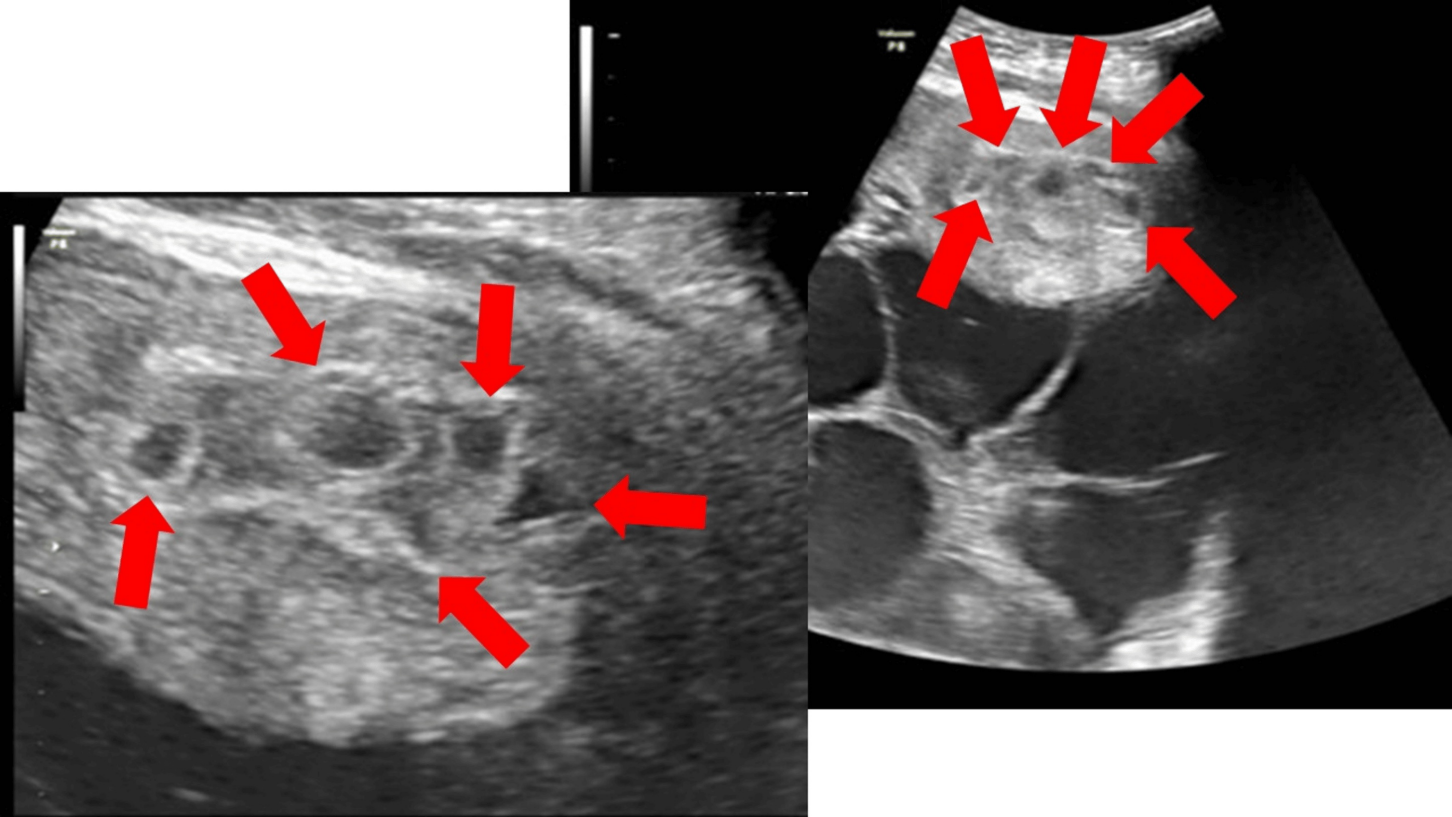
Transabdominal sonogram showing five gestational sacs within the uterine cavity at the time of diagnosis of a quintuplet pregnancy

On the sixth day of hospitalization (six weeks and two days of gestation), her hCG level had risen to 15,000 mIU/mL. A transabdominal ultrasound revealed five gestational sacs within the uterine cavity, leading to a diagnosis of quintuplets (Figure 4).

On the same day, a uterine evacuation procedure was performed, with an assistant providing exposure of the enlarged vulva. Perineal edema drainage was performed simultaneously. Gross examination of the uterine contents revealed only blood clots and endometrial tissue. The subsequent pathological diagnosis showed no evidence of mole or malignancy (chorionic villi positive, decidua positive).

**Figure 5:**
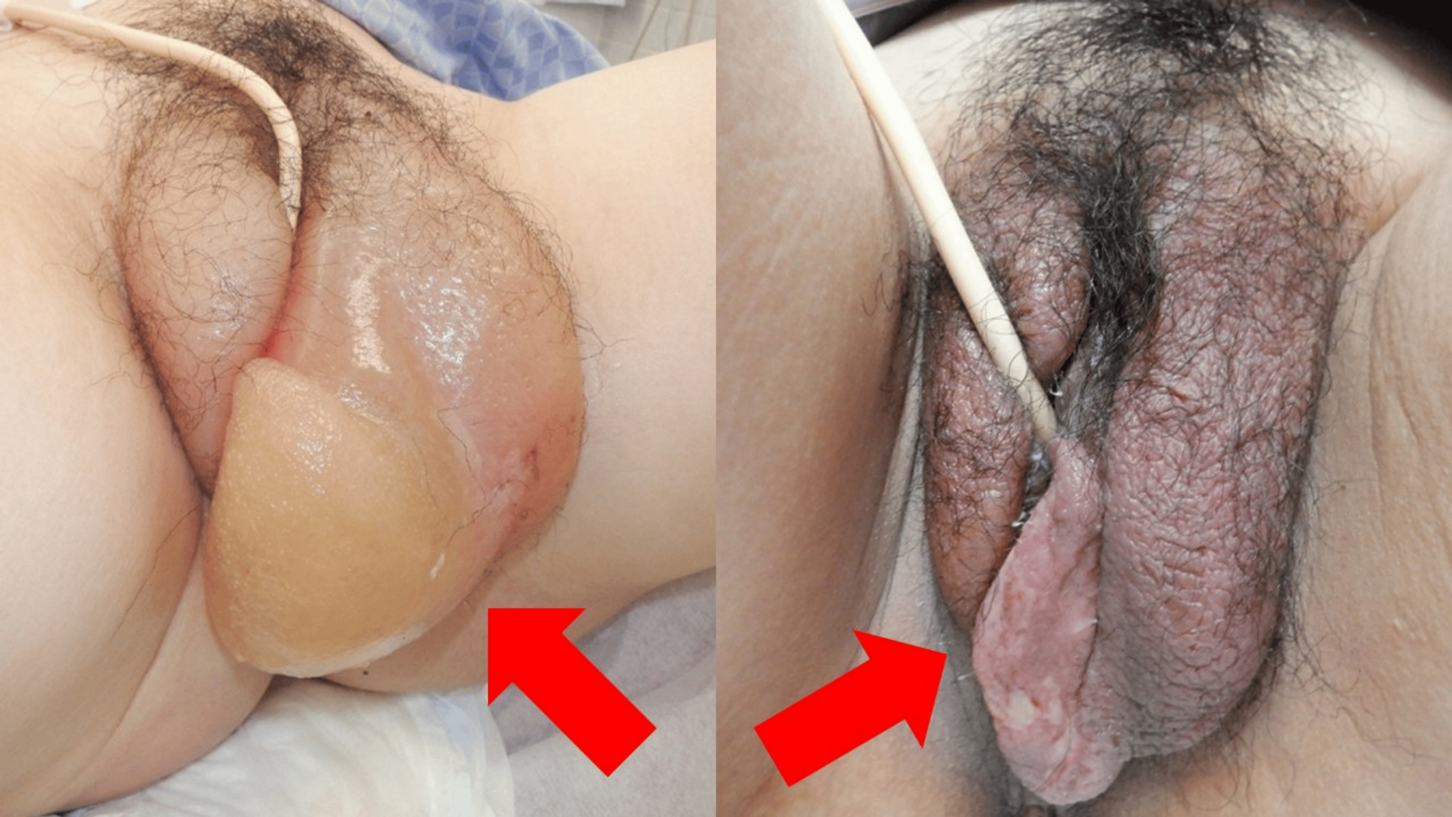
Clinical photographs of the vulva on admission (left) and on day 11 of hospitalization (right) Left image: Severe vulvar edema due to fluid extravasation, causing dysuria and impeding transvaginal ultrasound transducer insertion. Right image: Resolution of vulvar swelling following pregnancy termination and drainage of edema.

Following the procedure, she was monitored closely. Her respiratory status and vulvar edema improved (Figure 5), and she was transferred to the general ward on day 11 of hospitalization.

**Figure 6:**
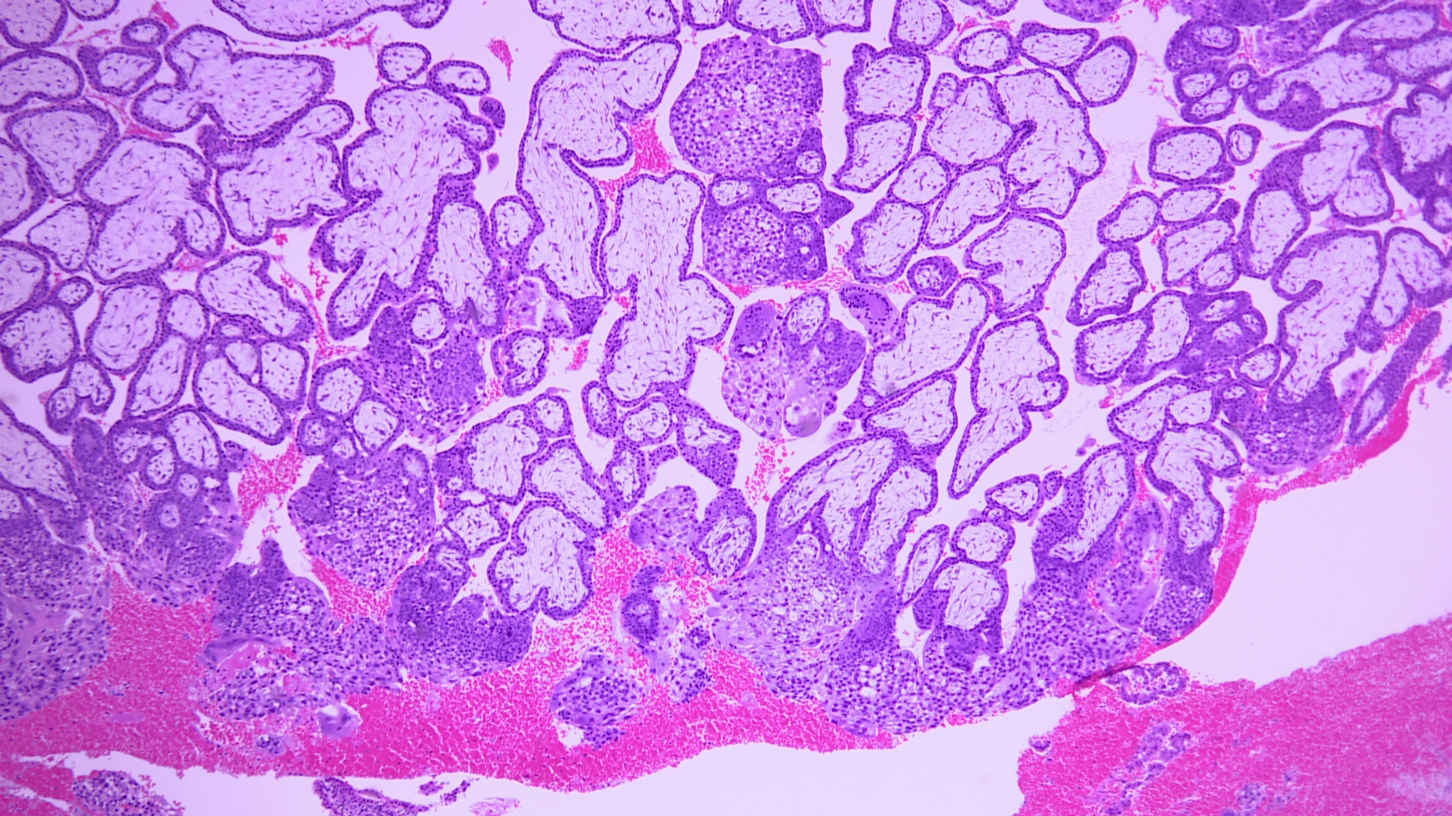
Pathological specimen confirming no evidence of mole or malignancy (chorionic villi positive, decidua positive)

The subsequent pathological diagnosis confirmed no evidence of mole or malignancy (chorionic villi positive, decidua positive) (Figure 6).

Conservative management was continued, and she was discharged on day 25. Her hCG level became negative 11 days after discharge, although both ovaries remained enlarged at 10 cm. Ovarian enlargement resolved by 39 days post-discharge, and menstruation resumed by 49 days post-discharge, at which point she was cleared to restart fertility treatment. A total of 91 days elapsed from the initial diagnosis to the planning of subsequent treatment.

## Discussion

Pathogenesis and diagnostic criteria of OHSS

The pathophysiology of OHSS is complex. It is primarily driven by ovarian release of VEGF, induced by hCG, which increases vascular permeability. This leads to leakage of intravascular fluid into the extravascular “third space” (mainly the peritoneal and pleural cavities) [1], causing ascites, pleural effusion, hemoconcentration, increased risk of thrombosis due to hypercoagulability, electrolyte imbalances, and impaired renal function from reduced circulating plasma volume [2,3]. The classification established by Golan and Weissman, categorizing OHSS as mild, moderate, or severe, is widely used [4]. Additionally, the American Society for Reproductive Medicine (ASRM) guidelines include a “critical” category for the most severe cases [5]. This case was classified as critical due to massive hydrothorax and acute renal failure. OHSS can also be classified by its time of onset: early-onset OHSS, caused by exogenous hCG and occurring within seven to eight days of administration, and late-onset OHSS, caused by endogenous hCG from the placenta in pregnancy, occurring 14-16 days or more after the initial hCG trigger [6]. Late-onset OHSS tends to be more severe and prolonged [7]. Our patient presented with features of both types, with initial symptoms appearing three days after the hCG trigger and severely worsening following pregnancy. Consequently, the duration of treatment was longer than typically seen for late-onset OHSS alone.

Risk factors and predictive factors for treatment

Several risk factors for OHSS are well established, including age under 35, Black ethnicity [5], PCOS, AMH levels >3.36 ng/mL [8], an antral follicle count ≥24 [9], and estradiol levels >3,500 pg/mL [5].

Factors predictive of treatment response include Hb levels, platelet counts, albumin levels, and fibrinogen levels. These markers reflect the pathophysiological hallmarks of OHSS, such as hemoconcentration, hypercoagulability, and hypoalbuminemia, and are crucial for monitoring recovery [3]. Furthermore, because hCG has an approximately 24-fold longer duration of action than LH, it induces prolonged luteal stimulation with supraphysiological levels of estradiol and progesterone, increasing the likelihood of OHSS [10]. Therefore, monitoring hCG trends is also essential. In this case, the decline in hCG levels post-termination correlated with the patient’s symptomatic improvement.

Importance of multidisciplinary treatment

While reports indicate a median recovery time of 11 days for pregnant patients with moderate to severe OHSS [3], our patient required 30 days from the first positive hCG test to discharge. Extensive evidence-based guidelines from the European Society of Human Reproduction and Embryology (ESHRE) and ASRM exist for the prevention of OHSS [5,11]; however, there is a lack of large-scale clinical trials and standardized treatment protocols for established severe OHSS. Some small studies suggest the utility of albumin administration [12], and that paracentesis may shorten hospital stays, improve pregnancy rates, and lower miscarriage rates, although the evidence is limited [5].

Once severe OHSS develops, patient-tailored therapy addressing circulatory, respiratory, and symptomatic changes is required, as demonstrated in our case. This underscores the importance of collaboration with the ICU. From an obstetrician-gynecologist’s perspective, it is essential to communicate predictors of prolonged severity with the critical care team daily.

While many cases of severe OHSS can be managed with pregnancy continuation, and one report suggests a significantly higher live birth rate compared to non-OHSS groups (88.9% vs. 73.5%) [7], termination of pregnancy should not be delayed when maternal life is at risk, as in our patient’s situation. Furthermore, although evidence for each individual intervention is limited, our experience suggests that a combined multidisciplinary approach may be highly effective for critical OHSS.

Quintuplets

Reports of quintuplets are currently limited to case reports. Although an older publication, one study tracked hCG progression in pregnancies up to quadruplets [13], reporting that hCG levels are approximately 15,000 mIU/mL when four gestational sacs are visible. Therefore, in cases of extremely elevated hCG levels, the possibility of a high-order multiple pregnancy must be considered, and termination of pregnancy should be discussed as an option.

## Conclusions

We managed a case of critical OHSS associated with a quintuplet pregnancy. The duration of hospitalization and time to resumption of menses were prolonged, significantly delaying the patient’s return to fertility treatment. While prevention is paramount, this case highlights the potential utility of a multidisciplinary approach in collaboration with critical care physicians for the treatment of critical OHSS. Decisions regarding pregnancy termination must be made promptly based on the mother’s systemic condition. Further reports are needed to establish effective multidisciplinary treatment protocols aimed at achieving the fastest possible symptom resolution and shortening the time to resumption of subsequent fertility treatments.
